# Severe Osteoporosis With Pathogenic *LRP5* Variant

**DOI:** 10.1210/jcemcr/luae021

**Published:** 2024-02-23

**Authors:** Felicity Stringer, Natalie A Sims, Nirupa Sachithanandan, Jasna Aleksova

**Affiliations:** Department of Endocrinology, St Vincent's Hospital Melbourne, Fitzroy, Melbourne, VIC 3065, Australia; St Vincent's Institute of Medical Research, Fitzroy, Melbourne, VIC 3065, Australia; Melbourne Medical School, The University of Melbourne, Parkville, VIC 3052, Australia; Department of Endocrinology, St Vincent's Hospital Melbourne, Fitzroy, Melbourne, VIC 3065, Australia; Melbourne Medical School, The University of Melbourne, Parkville, VIC 3052, Australia; Department of Endocrinology, St Vincent's Hospital Melbourne, Fitzroy, Melbourne, VIC 3065, Australia; Department of Medicine, Monash University, Clayton, VIC 3168, Australia; Centre for Endocrinology and Metabolism, Hudson Institute of Medical Research, Clayton, VIC 3168, Australia

**Keywords:** osteoporosis, *LRP5*, Wnt signaling pathway, romosozumab

## Abstract

A 24-year-old female patient was diagnosed with osteoporosis after presenting with numerous fractures throughout her childhood and adolescence. Risk factors included chronic constipation, severe vitamin D deficiency, and long-term high-dose steroid use for severe eczema. Metabolic bone disorder clinical exome screening (limited panel of metabolic bone disorders and gastrointestinal disorders) was undertaken and revealed a class 4 likely pathogenic variant in the *LRP5* gene known to cause osteoporosis. Optimal treatment for patients with this variant is not well defined. A literature review of the condition and potential treatment options is discussed.

## Introduction

The Wnt signaling pathway plays a critical role in bone metabolism, and the LRP5 receptor is an essential component of this pathway. We describe the case of a young female patient with severe osteoporosis, who was found to have a class 4 likely pathogenic heterozygous variant in the *LRP5* gene, and we discuss the optimal treatment options currently available, and future potential therapeutic strategies.

## Case Presentation

A 24-year-old female patient presented with more than 30 fractures throughout her childhood and adult life. These included clavicular, metatarsal, radial, ulnar, and scaphoid fractures. She had no femoral, humeral, or vertebral fractures.

Her past medical history was significant for chronic constipation identified in early infancy. She previously had a cecostomy tube and multiple electronic stimulations with no benefit. She is currently managed with daily polyethylene glycol iso-osmotic purgative via a nasogastric tube. Other past medical history includes chronic eczema with high-dose prednisolone (25–50 mg daily) for 6 years. The patient also had obesity, with a body mass index (BMI) of 56, with adverse responses to very low-energy diets (including hypokalemia and allergies), and limited response to 2 years of phentermine therapy; financial restrictions prohibited commencement of a glucagon-like peptide 1 analogue. She had no history of visual impairment, and she reported no new visual symptoms.

At presentation, her medications were prednisolone 25 mg daily, polyethylene glycol iso-osmotic solution 2 L daily, topiramate 50 mg twice daily, and an implanon in situ for management of irregular menstrual cycle and contraceptive use. She was a nonsmoker, with no family history of osteoporosis or fractures.

## Diagnostic Assessment

A secondary osteoporosis screen revealed vitamin D deficiency (6.8 ng/mL [17 nmol/L], normal reference range: > 20 ng/mL [> 50 nmol/L]) but no other underlying abnormalities (see Table 1). Her bone density at the wrist was normal (Z-score +0.2); however, her high BMI precluded dual-energy x-ray absorptiometry (DXA) imaging at the spine and hip.

**Table 1.** Investigation results at presentation

	At presentation	Reference range
Adjusted calcium	10.18 mg/dL (2.54 mmol/L)	8.42–10.42 mg/dL (2.10–2.60 mmol/L)
Phosphate	4.22 mg/dL (1.36 mmol/L)	2.32–4.65 mg/dL (0.75–1.50 mmol/L)
eGFR	> 90 mL/min/1.73m²	
Creatinine	0.68 mg/dL (60 umol/L)	0.51–1.02 mg/dL (45–90 umol/L)
Vitamin D	**6.8 ng/mL** **(17 nmol/L)**	> 20 ng/mL (>50 nmol/L)
HbA1c	5.2% (33 mmol/mol)	4.0%–6.0% (20–42 mmol/mol)
Bone density scan GE Lunar	Right forearm BMD is 0.909 grams/cm² Z-score +0.2, T-score +0.2	
Thoracolumbar x-ray	Nil fractures	

Abnormal values are shown in bold font. Values in parenthesis are International Systems of Units (SI). Abbreviations: BMD, bone mineral density; eGFR, estimated glomerular filtration rate; HbA1c, glycated hemoglobin.

## Treatment

Initial management included diet optimization and restoration of serum vitamin D. Given her significant fracture history and risk factors, zoledronic acid was administered. However, this dose was complicated by significant extravasation and subsequent inpatient admission with cellulitis requiring intravenous antibiotics, and no further doses have been administered.

The patient consented for metabolic bone disorder clinical exome screening (limited panel of metabolic bone disorders and gastrointestinal disorders, conducted at the Australian Genome Research Facility). This revealed a previously unreported monoallelic class 4 likely pathogenic variant in the *LRP5* gene (c.1307delG p.(Gly436Alafs*7)). This is a frameshift variant, predicted to cause a premature stop codon, and was considered likely arising de novo. After expert discussion, the consensus was that romosozumab treatment would be optimal for her bone health.

## Outcome and Follow-Up

Re-engagement and review by Dermatology led to a reduction in prednisolone to 7.5 mg daily, and bariatric review has allowed commencement of hospital-funded semaglutide for weight loss. Vitamin D restoration was initially successful, with vitamin D levels improving to 26.4 ng/mL (66 nmol/L). However, more recently, her vitamin D levels have decreased to 16.8 ng/mL (42 nmol/L). A further intramuscular dose of vitamin D has been arranged prior to commencement of romosozumab.

## Discussion

The canonical Wnt signaling pathway plays a critical role in bone metabolism (1-3). The Wnt ligand forms a complex with 2 receptors on the cell surface (Frizzled [Fzd] and LRP5 or LRP6) and stabilizes beta catenin by inhibiting its phosphorylation; this promotes its translocation to the nucleus and transcription of Wnt-responsive genes, including *RUNX2* (an osteoblast commitment gene) and *TNFRSF11B* (the gene for the osteoclast inhibitor osteoprotegerin) (3). This has a 2-pronged effect, promoting the progression of skeletal stem cells from osteoblastic precursor cells into mature osteoblasts and suppressing osteoclast differentiation (1-3).

Osteoporosis-pseudoglioma syndrome (OPPG) is a rare autosomal recessive disorder of severe juvenile osteoporosis and congenital blindness, caused by homozygous loss-of-function in the *LRP5* gene (3, 4). Heterozygous *LRP5* loss-of-function variants, as seen in this case, cause juvenile-onset osteoporosis and familial exudative vitreoretinopathy. Although a less severe phenotype than OPPG, heterozygous loss-of-function variants are also associated with increased fractures from childhood, and increased risk of blindness due to premature arrest of the retinal vasculature (3, 4). Patients with early-onset osteoporosis (premenopausal women and men < 50 years) have been shown to have high rates of *LRP5* variants, with reported prevalence of 8% to 20% in this cohort (2, 5). There have not been associations with severe eczema or colonic dysmotility with these variants, as experienced in this case.

Treatment for patients with *LRP5* pathogenic variants is not well established. In these individuals, bisphosphonates, denosumab, and teriparatide have all been reported to improve bone mineral density (BMD) as measured on DXA scans and to reduce fracture risk in case series. Currently there is the most evidence for safety and efficacy using bisphosphonates (6-8).

Romosozumab is an anabolic therapy for postmenopausal osteoporosis (9). It is a humanized monoclonal neutralizing antibody to sclerostin, an antagonist of the Wnt pathway. Sclerostin inhibits bone formation by preventing the engagement of LRP5/6 with Fzd (10). It was proposed that the anabolic actions of romosozumab may be less effective in patients with OPPG due to loss of function of *LRP5*, since they already lack the LRP5/Fzd binding that sclerostin inhibits (11). However, *LRP5*-deficient mice responded to anti-sclerostin treatment with increased bone formation, trabecular bone mass, cortical bone mass, and BMD (11). The authors concluded that in the absence of the LRP5 receptor, the anabolic effects of sclerostin depletion may occur via their inhibition of the Wnt interaction with other receptors (such as LRP4 or LRP6). Since patients with *LRP5* variants may also be more reliant on LRP4 and LRP6, by increasing the activity of the Wnt pathway via LRP4 or LRP6 these patients may have significant gains in bone mass.

Neutralizing antibodies targeting the LRP4 and LRP6 receptors and other Wnt antagonists, such as DKK1, bispecific anti-sclerostin, and DKK1 antibodies, as well as Wnt1 activation, are alternative approaches that have been developed and shown to increase bone mass in mouse models (12-15). This is a promising research area that requires further exploration to determine their potential therapeutic benefit in humans, either in combination with romosozumab or as alternatives.

We have described a complex clinical case of likely multifactorial osteoporosis, contributed to by an uncommon pathogenic variant of the *LRP5* gene and subsequently the Wnt signaling cascade. However, the patient's long-term high-dose prednisolone is a major contributing factor, and ongoing dose reduction continues to be a focus of management. Further data beyond case reports would bring more clarity to the best management strategies for patients with *LRP5* variants.

## Learning Points

- The Wnt signaling pathway plays a critical role in bone remodeling and is the target for anti-osteoporosis therapy (romosozumab).
- We describe a novel, likely pathogenic variant in the *LRP5* gene that has resulted in multiple fragility fractures at a young age.
- The optimal therapy for patients with variants in the *LRP5* gene causing severe bone fragility and osteoporosis is yet to be determined, with evidence limited to case reports. Most studies have shown safety and efficacy for bisphosphonates.
- Romosozumab may be beneficial in these patients, given alternate receptors involved in the Wnt signaling pathway, and when the variant is heterozygous.

## Contributors

All authors made individual contributions to authorship. F.S. and J.A. were involved in clinical care of the patient. F.S. wrote the case report and body of the discussion. J.A., N.A.S., and N.S. made revisions and improvements to the article. All authors approved the final copy of the article and application to the journal.

## Data Availability

Data sharing is not applicable to this article as no datasets were generated or analyzed during the current study.

## Abbreviations

BMD: bone mineral density
BMI: body mass index
DXA: dual-energy x-ray absorptiometry
OPPG: osteoporosis-pseudoglioma syndrome
